# Wood sawdust waste-derived nano-cellulose as a versatile reinforcing agent for nano silica cement composites: a systematic study on its characterization and performance

**DOI:** 10.1038/s41598-023-39788-x

**Published:** 2023-08-07

**Authors:** Amr H. Badawy, M. S. El-Feky, A. Maher El-Tair, Mohamed Kohail

**Affiliations:** 1 Department of Civil Engineering, National Research Centre, Giza, Egypt; 2 Structural Engineering Department, German University in Cairo, Cairo, Egypt; 3 Structural Engineering Department, Faculty of Engineering, Ain Shams University, Cairo, Egypt

**Keywords:** Civil engineering, Nanoparticles

## Abstract

The development of sustainable construction materials is a pressing concern for researchers worldwide, as the cement industry is a major contributor to environmental degradation. The incorporation of nano-materials with cement composites has emerged as a promising solution to sustainable materials production. In this study, the effect of the addition of nano cellulose produced from wood sawdust waste on the performance of cement-based nano-silica composite was investigated. The nano-materials were incorporated at low concentrations and in gel form to eliminate the need for any advanced dispersion techniques. The results indicated that the addition of even low concentrations of nano cellulose significantly enhanced the compactness and mechanical properties of the cement matrix. The crack propagation was observed to be arrested with better adherence to the cement hydration product, which resulted from the presence of nano-silica. The nano cellulose fibers were found to bridge the calcium silicate hydrate products, arresting the propagation of cracks at their initial condition. The high pozzolanic reactivity of nano-silica ensured a minimal amount of calcium hydroxide, which is a significant contributor to the carbon footprint of cement production. Overall, the findings of this study suggest that the incorporation of nano cellulose from wood sawdust waste with cement-based nano-silica composite can lead to the development of sustainable and high-performance building materials with improved mechanical properties and reduced environmental impact.

## Introduction

The construction industry is a significant contributor to environmental pollution and waste generation, and reducing the consumption of non-biodegradable materials and minimizing waste is crucial for a cleaner environment. To achieve this, researchers have investigated the use of supplementary cementitious materials (SCMs), including micro and nano-scale materials, to partially replace cement in concrete. While various types of fibers have been used to improve the ductility and brittleness of cement mortars and concrete, natural fibers such as cellulose nano-fibers have gained attention due to their cost-effectiveness and environmental friendliness.

Various types of micro and nano-scale SCMs, including Fly Ash^1–8^, ground-granulated blast furnace slag (GGBS)^9–12^, and metakaolin (MK)^13–24^, nano-clay (NC)^25–29^, carbon nano-tubes (CNT)^30–34^, nano-cellulose (NCL)^35–39^, and nano-titanium^40–42^ have been studied as partial replacements for cement in concrete and cement mortars.

However, the use of traditional fibers such as carbon and polymer fibers is limited by their high cost, low bond strength, and low corrosion resistance, while glass fibers have low bond strength with cement and low alkaline resistance. Therefore, natural fibers such as cellulose nano-fibers have gained attention due to their cost-effectiveness and environmental friendliness^43–51^.

Nano-cellulose can be extracted from plants, animals, and bacteria^52,53^. Nano-cellulose is characterized by its stiffness, lightweight, availability, and environmentally friendly. Previous studies have shown that cellulose nano-fibers can enhance the flexural strength, toughness, and impact resistance of concrete, but there are still challenges related to compressive strength^54–62^.

Researchers have investigated the use of bacterial and raw and sonicated nano-cellulose on the mechanical properties and microstructure of cement mortars and paste. Akhaghi et al.^63^ studied the effect of using bacterial NCL on the cement mortars; bacterial NCL was added as a powder and gel with ratios of 0.1, 0.3, and 0.5 wt%. The results indicated that using bacterial NCL as a gel had a good impact on the mechanical properties of the cement mortars than using NCL as a powder, where the flexural strength was improved at NCL powder was 0.5% while at NCL gel was 104% than that of the control mix. At the same time, the water absorption was reduced by 26% in the powder and 37% in the gel^63^. Cao et al.^64^ studied the effect of raw and sonicated NCL on the microstructure of cement paste. The results indicated that the porosity was reduced to 14.8% and 14.4% for raw and sonicated NCL. This reduction indicates increasing the degree of hydration and improving the microstructure of the cement paste^64^.

The coupling effect of nano-silica and nano-cellulose has also been studied in previous research^38^ However, there is a lack of research on the interaction between cellulose nano-fiber produced from wood sawdust waste and nano-silica hybrid cement, and its effect on cement hydration.

Therefore, this study aims to investigate the effect of cellulose nano-fiber produced from wood sawdust waste and nano-silica hybrid cement on cement hydration. The study examines the effective interaction between the matrix and fiber to increase the productivity and performance of cement composites. To avoid agglomeration, nano-silica and nano-cellulose were added without applying any means of dispersion. The study investigates compressive strength, absorption, and abrasion resistance, and analyzes the microstructure of the cement matrices using Scanning Electron Microscopy, X-Ray Diffraction, and Thermogravimetric Analysis.

The main aim of the study is to solve two crucial concerns in the construction industry: lowering the consumption of non-biodegradable materials and minimizing waste, based on the work presented in this introduction and the gaps discovered in the prior research. The study's main objective is to replace some of the cement in concrete and cement mortars with nanomaterials, notably nano-silica and nano-cellulose made from waste wood sawdust. One of the significant contributions of this research is that it investigates the effective interaction between the matrix and fiber, which is essential for improving the productivity and performance of cement composites. moreover, there is not any method of dispersion used in this investigation, and low dosages of both nano-cellulose and nano-silica are used as well. In addition, a cutting-edge method of combining these materials into construction applications is the utilization of nano-cellulose in gel form. The research investigates the compressive strength, absorption, and abrasion resistance of the cement matrices as well as their microstructure through SEM, XRD, and TGA. finally, the research is considered unique in its focus on using waste materials and nanomaterials to develop high-performance, sustainable building materials which have the potential to revolutionize the construction sector and reduce its environmental effect.

## Experimental program

### Materials

Ordinary Portland cement (CEM I) with a grade of 42.5 N, complying with ASTM C150^65^, was used. Table 1 shows the chemical composition of the used cement. Natural sand was used, complying with ASTM C33^66^ with a particle size less than 0.5 mm, and specific gravity of 2.89 g/cm^3^. Table 2 shows the properties of the fine aggregate used. Nano-silica with an average particle size of 30 nm was used. The chemical properties of nano-silica are shown in Table 1. Nano-Cellulose was provided by a local company in Egypt called NCTECH^®^. The used NCL had a nanometric diameter of 4–12 nm and a micrometric length of 100–450 nm, with a specific surface area of 80 m^2^/gm. According to NCTECH^®^, NCL was extracted from wood sawdust. The extraction process of NCL involves the removal of all impurities such as lignin, pectin, wax, and soluble sugar. NCL was provided as a fully dispersed gel with 5% NCL.

**Table 1:** Chemical composition of cement, and nano-silica.

	SiO_2_	Fe_2_O_3_	Al_2_O_3_	CaO	MgO	TiO_2_	Na_2_O	K_2_O	P_2_O	L.O.I
Cement	20.13	3.61	5.32	61.63	2.39	–	0.37	0.13	–	1.96
NS	99.1	0.06	0.13	0.14	0.11	–	0.4	–	0.01

**Table 2:** Physical properties of sand.

Property	Sand
Specific weight (g/cm^3^)	2.89
Bulk density (kg/m^3^)	1.67
Fineness modulus	2.75
Water absorption %	–
Crushing value %	–
Clay and fine dust content %	1.95

### Mix design proportions, sample preparation, and curing

In this research program, 16 cement composites mixes were used with a total of 192 specimens; control mix, three mixes using nano-cellulose with an addition ratio from the weight of cement; 0.5%, 0.75%, and 1%, and three mixes using nano-silica alone from the cement weight with addition ratios; 0.5%, 1.0%, and 1.5%. In addition, nine mixes were used as a combination between nano-silica and nano-cellulose to study the combined effect. Table 3 shows the mixes compositions.

**Table 3:** Experimental matrix for cement mortar with NCL and NS.

Mixture ID	Sand	Cement	NCL	NS	Water	SP
	(gm)	(gm)	%	%	(gm)	(gm)
C	3000	1000	0	0	450	20
0.5NCL-0NS	3000	1000	0.5	0	450	20
0.75NCL-0NS	3000	1000	0.75	0	450	20
1.0NCL-0NS	3000	1000	1.0	0	450	20
0NCL-0.5NS	3000	1000	0	0.5	450	20
0NCL-1.0NS	3000	1000	0	1.0	450	20
0NCL-1.5NS	3000	1000	0	1.5	450	20
0.5NCL-0.5NS	3000	1000	0.5	0.5	450	20
0.5NCL-1.0NS	3000	1000	0.5	1.0	450	20
0.5NCL-1.5NS	3000	1000	0.5	1.5	450	20
0.75NCL-0.5NS	3000	1000	0.75	0.5	450	20
0.75NCL-1.0NS	3000	1000	0.75	1.0	450	20
0.75NCL-1.5NS	3000	1000	0.75	1.5	450	20
1.0NCL-0.5NS	3000	1000	1.0	0.5	450	20
1.0NCL-1.0NS	3000	1000	1.0	1.0	450	20
1.0NCL-1.5NS	3000	1000	0.01	1.5	450	20

For nano-silica mixes, nano-silica was added as a powder to cement and mixed dry for 2 min. While for nano-cellulose, it was added to the first half of mixing water and added to the dry mix and continued mixing for another 2 min. Superplasticizer was added to the second half of the mixing water and then added to the mix and continued mixing for another 3 min. Finally, sand was added to the mix and continued mixing for another 3 min. After the mixing process had finished, the mortar had cast into the molds. Then, the molds were placed on the vibrator to compact the specimens fully. The casted specimens were placed for 2 h after casting until the final setting of the cement mortar. Then the specimens were subjected to water curing until the day of testing.

### Test methods

Compressive strength results were the average of three 5 × 5 × 5 cm samples per each mix, the test was applied using a universal testing machine 1000 KN at a loading rate of 0.5 N/mm^2^ s after 7 and 28 days of curing. In order to evaluate the durability of the mixes at the hardened state, the water absorption test and the abrasion tests were performed after 28 days of curing. Compaction and curing of all specimens were executed according to the recommendations of ASTM C31. Microstructural properties of the samples were determined through scanning electron microscope (SEM), thermogravimetric analysis (TGA), and X-ray diffraction (XRD).

## Results and discussions

### Compressive strength

**Figure 1:**
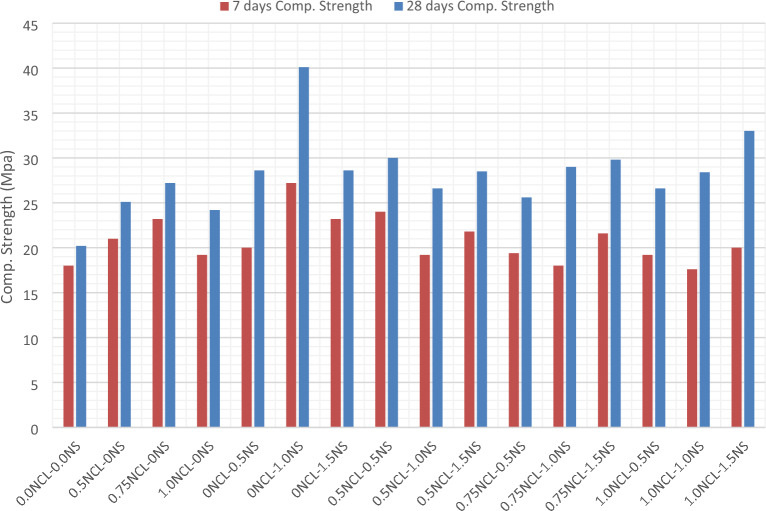
7- and 28-days compressive strength of NS and NCL cement mortars.

The compressive strength results for the cement mortar mixes after 7 and 28 days are shown in Fig. 1. After 7 days, the maximum compressive strength obtained was at using 1% NS of the cement weight alone with a strength gain 51% than the strength of the control mix. As all NS mixes had gain strength than that of the control mix. While when using NCL, all the mixes had gain strength similar to NS mixes but with a relatively lower gain percentage. In whish, the maximum gain obtained was at 0.75% NCL of the cement weight with 29% gain than that of the control mix.

This could be attributed to the effeciency of nano silica small addition even without sonication to better dispersion and consequently to increasing their pozzolanic reactivity over higher replacement ratios as previously reported in many research articles^67–69^. The higher pozzolanic reactivity not only increased the formation of more C–S–H, but also positively affected the densification of the matrix especially for the interfacial transition zone (ITZ) between the aggregates and the cement paste^70–72^. While in NCL mixes, the lower gain in strength after 7 days was due to the hydroxyl and carboxyl groups in the cellulose which react with Ca^+^ to form complexes which had a negative effect on the hydration reaction and resulting in the delaying of the setting time^73^. On the other hand, after 28 days, the absorbed water by NCL was released into the cement matrix, which had a great role in continuing un-hydrated cement particles in which the mechanical properties and the microstructure of NCL mixes had improved^74,75^.

When investigating the combined effect of NCL and NS, generally, the addition of NCL with small ratios had a negative effect on the strength than that of NS mixes alone, but still higher than that of the control mix, except for mix 1.0% NCL and 1.5% NS. The addition of NCL to NS without sonication had a negative effect on the hydration reaction of NS due to the agglomeration that occurred to NS and NCL particles together, besides the improper dispersion of cellulose nano-fibers in the mix. The agglomeration of NCL particles will reduce the specific surface area of NS available to work as extra nucleation spots and, therefore, reduce NS’s reactivity and lower the strength.

### Absorption test

**Figure 2:**
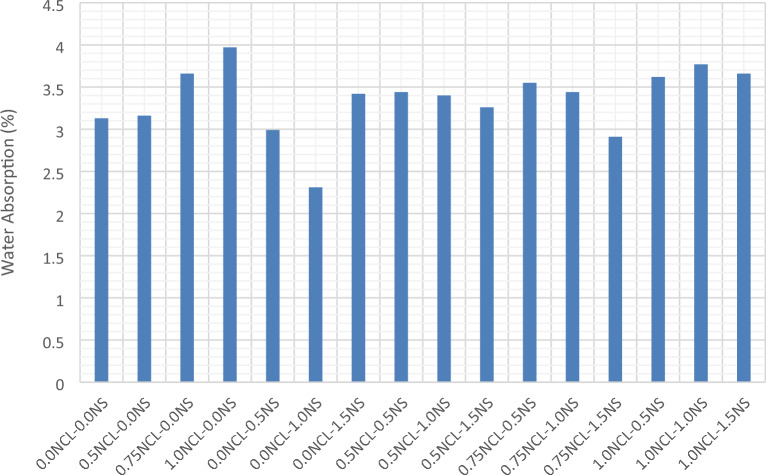
Water absorption test results for NS and NCL cement mortars.

The water absorption is due to the capillary action of the cement matrix. The water absorption test showed the same trend as in compressive strength results. As the compressive strength improved, the water absorption was reduced. In other words, the factors that affect the strength of the mixes were the same factors that reduced the water absorption property for the mixes. From the results shown in Fig. 2, the highest compressive strength obtained for mix with NS only at 1% of cement weight, was also had the lowest water absorption value than the control mix. However, when adding 1.5% NS the water absorption test was increased than that of the control mix. This can be attributed to the agglomeration that occurred to the silica nano-particles due to the high percentage of NS added without subjected to sonication prior adding to the mix^16,19,76–78^. While for NCL mixes, the water absorption had increased due to improper dispersion and thus agglomeration, resulting in a weak bond between cellulose nano fibers and the cement matrix, which initiates voids and thus increases the porosity of the mix and increases the water absorption^63,79^. On the other hand, mixes containing NS and NCL had also increased the water absorption property than the control mix but with a narrow limit, except for mix with 0.75 wt% NCL and 1.0 wt% NS as the water absorption was reduced by 7%. This can be attributed to the pozzolanic, nucleation sutes, and the filling effects of the silica nano particles that helped improving the microstructure and strength of the cement matrices and significantly reducing the number of voids formed as a result of the agglomerated cellulose nano fibers and their irregular arrangement in the cement matrices.

### Abrasion test

**Figure 3:**
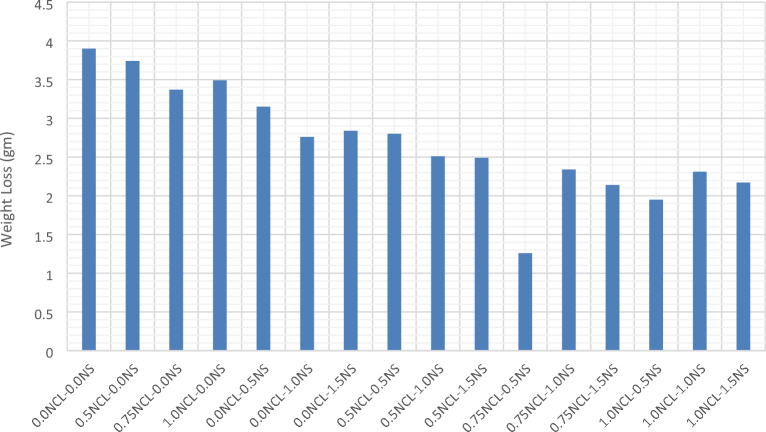
Abrasion resistance test results for NS and NCL cement mortars.

From Fig. 3, The addition of both nano-silica and cellulose nano-fibers had improved the cement mortar’s abrasion resistance, whether by adding NS or NCL alone or with a combination of them. The pozzolanic and the nucleation of nano-silica had improved the microstructure of the cement mortars to different degrees. These findings will be confirmed later in the microstructure analysis investigation. While cellulose nano-fibers had improved the crake propagation and the tensile strength of the cement matrix and improved the hydration process and produced more CSH^80^. The mix with 0.5 wt% of NS and 0.75 wt% NCL obtained the optimum results with an improvement of 68% than that of the control mix.

When comparing these results with the results obtained by El-Tair et al.^70^, nano-silica and cellulose nano-fibers were added to the mix after being subjected to sonication and with a higher dosage than that done in this research. And all the results obtained were with an improvement in the mechanical and durability properties of the produced cement composites. However, in this research, nano-silica and cellulose nano-fibers were added in smaller percentages without sonication. In addition to the results obtained by El-Feky et al.^38^, nano-cellulose and nano-silica were subjected to sonication before adding to the mix. From this, sonication is the key reason for obtaining a better performance for any nano-material in the cement matrix by improving its dispersion in the cement matrix and thus obtaining a homogeneous microstructure.

### Microstructure tests

#### SEM

**Figure 4:**
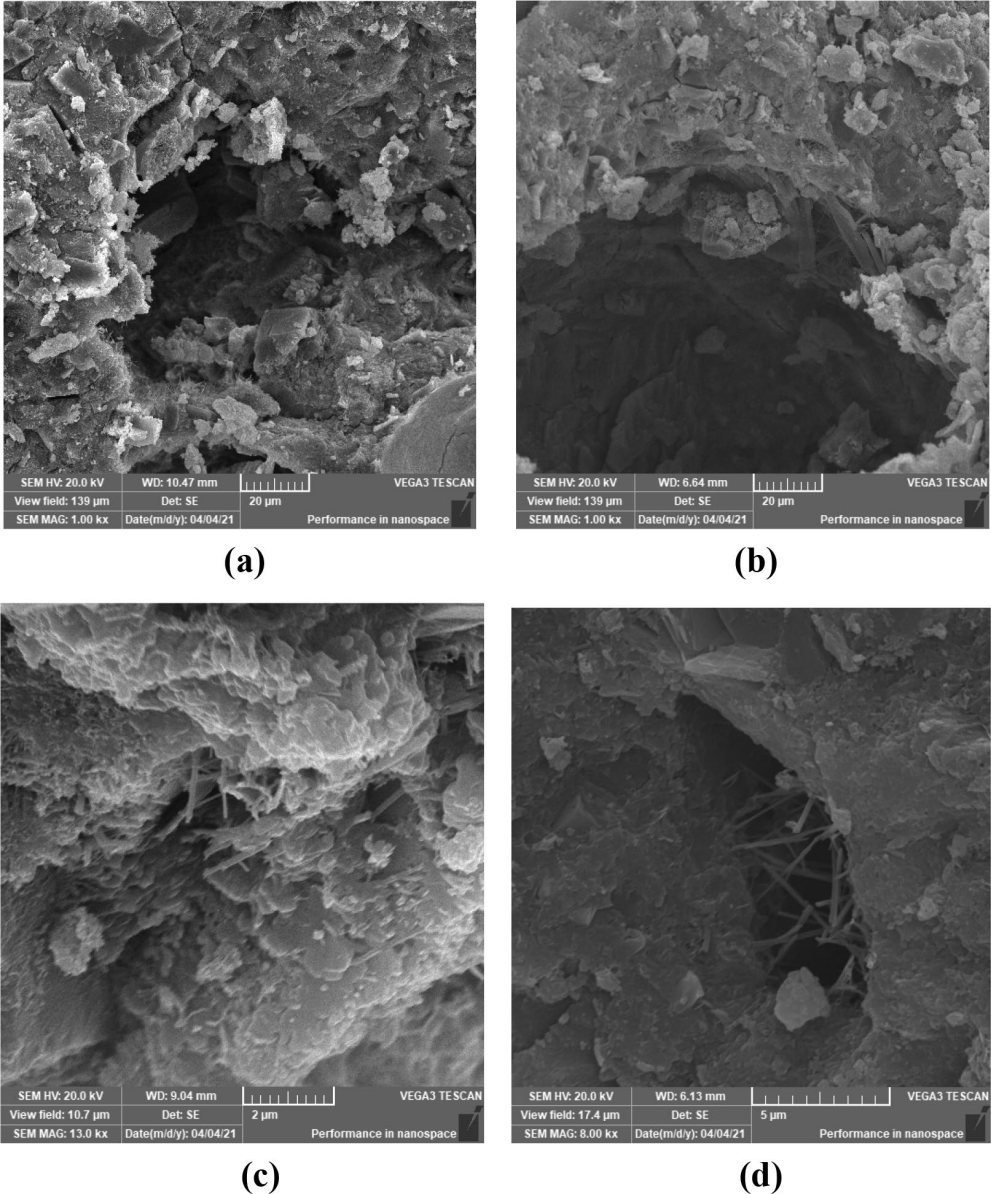
Scanning electron micrographs of (**a**) control specimen; (**b**) specimen with 0% NCL and 1.5% NS; (**c**) specimen with 1% NCL and 0% NS; (**d**) specimen with 1% NCL and 1.5% NS.

Generally, the cement matrix for the control mix (mix without any nano addition), found to be less dense than the matrices with the nano additions, the calcium silicate hydrate as well as the calcium hydroxide crystals can be well identified. Therefore, voids of relatively large size could be indicated in this matrix. As for the matrix incorporated nano-silica particles, the filling effect of the nano-silica helped densify the matrix, as seen in Fig. 4. In addition, the SEM micrograph showed the spread of the calcium silicate hydrate products over the calcium hydroxide crystals. While from the SEM micrograph of the matrix incorporated nano cellulose, the nano cellulose fibers could be easily identified reinforcing the calcium silicate hydrate products leading to a significant enhancement within the matrix strength as has been mentioned in the compressive strength section. In addition, the porosity had significantly enhanced, The reduced porosity and pore size could be attributed to the nano cellulose performance that at first absorbed water and then released this water to promote cement hydration, and consequently, producing more cement hydrates. Finally, for the matrix incorporated the hybrid nano-materials (nano-silica, and nano cellulose), the spread of calcium silicate hydrate products was identified with almost null of the presence of calcium hydroxide crystals indicating the high reactivity of the nano silica with the residual calcium hydroxide leading to denser highly stiffened matrix, moreover, the nano cellulose fibers was found bridging the calcium silicate hydrate products arresting the propagation of crack at its initial condition, the situation that represents the efficiency of the hybrid nano materials in enhancing the strength and durability of the cement matrices as the crack arresting will reduce the crack width and depth reducing the number of the main weak points for water or aggressive ions infiltration into the cement matrices. Similar findings were reported by a number of previous researches^36,64,81^.

#### XRD

In this study, the use of X-ray diffraction (XRD) to study the phase composition of cement-based materials was presented and discussed. XRD is a technique that uses X-rays to determine the arrangement of atoms in a crystal lattice, which can provide information about the chemical composition and crystal structure of a material.

**Figure 5:**
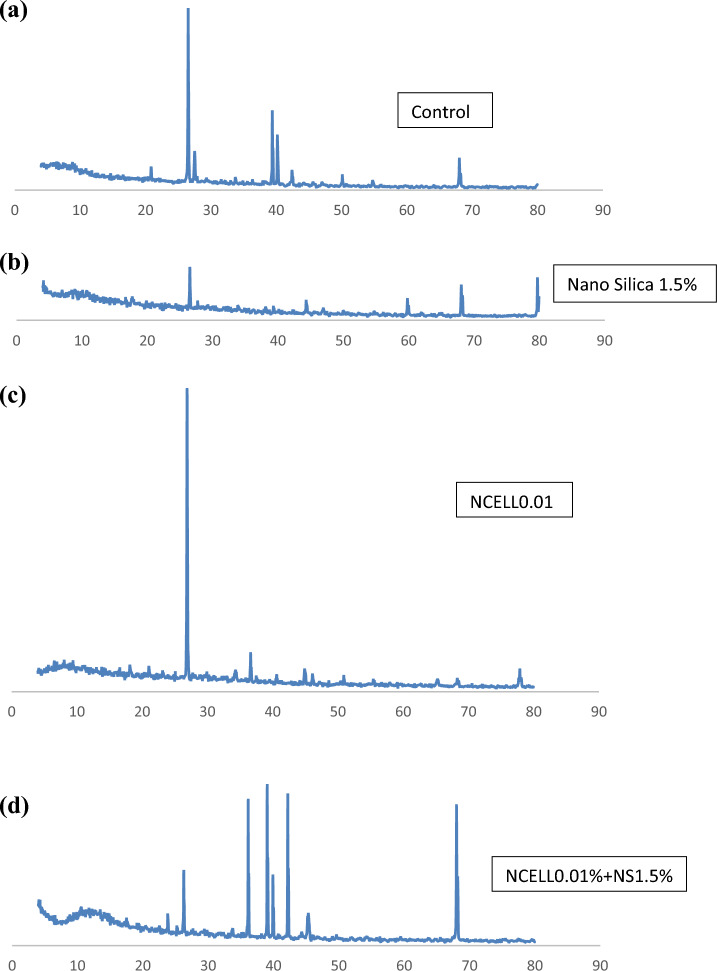
XRD analysis (**a**) control specimen; (**b**) specimen with 0% NCL and 1.5% NS; (**c**) specimen with 1% NCL and 0% NS; (**d**) specimen with 1% NCL and 1.5% NS.

The XRD spectra for the control mixture (without any nano additions), the mixture with the optimum content of nano-silica particles, the mixture with the optimum content of nano-cellulose particles, and the mixture with the optimum hybrid mixture are shown in Fig. 5. The peaks in the spectra at 18.00, 34.10, 47.12, and 50.81 are attributed to portlandite (P), which is a byproduct of cement hydration. The peaks in the 2θ region of 29–33 are due to the main components of non-hydrated cement particles, tricalcium silicate (C3S) and dicalcium silicate (C2S).

Statistically significant changes in peak values of crystalline phases, such as Ca(OH)_2_ and CaCO_3_, and the appearance of new peaks indicated changes in the phase composition of existing phases or the formation of new phases in the microstructure. It can be concluded from the figure that no significant difference was observed in the phase composition of the nano-cellulose-modified cement mixture compared to the control mixture, based on the XRD spectra. However, the peak of calcium hydroxide and the peak of non-hydrated cement were reduced by nano-silica compared to the control mixture.

The P-peaks of the cement mixture with nano-silica and NCell at 18:00 and 34:10 appear to be slightly smaller than the P-peaks of the control mixture. However, it should be readdressed that the XRD measurements are qualitative and care must be taken in the final conclusion. Finally, similar findings were reported by previous research studies that confirms what had been reported from the results^82–84^. Overall, the use of XRD to evaluate the effect of nano-silica and nano-cellulose on the phase composition of cement-based materials suggested that nano-silica reduced the presence of calcium hydroxide and non-hydrated cement in the mixture.

The diffraction spectra of the control mixture, the mixture containing the optimum content of the nano silica particles and the optimum content of the nano cellulose particles, and the mixture containing the optimum hybrid mixture are shown in Fig. 5. The (2θ) peaks at 18.00, 34.10, 47.12, and 50.81 are due to portlandite (P)^85^. The peaks in the 2θ region of 29–33 are due to the main components of non-hydrated cement particles, tricalcium silicate (C3S) and dicalcium silicate (C2S)^85^. The XRD results are useful for qualitatively studying the phase composition of cement-based materials. Statistically significant changes in peak values of crystalline phases such as Ca(OH)_2_ and CaCO_3_, and the appearance of new peaks indicate changes in the phase composition of existing phases or the formation of new phases in the microstructure. A comparison of the spectra of the cement mixture shows no new phase in the structure due to the addition of NCell within the resolution of the measurement. The P-peaks of the cement mixture with nano-silica and NCell at 18:00 and 34:10 appear to be slightly smaller than the P-peaks of the control mixture. However, keep in mind that XRD measurements are qualitative and care must be taken in the final conclusion. Therefore, it can be concluded from the XRD spectra that no significant difference can be observed in the phase composition of the NCell-modified cement mixture compared to the control mixture. The only exception was the peak of the mixture containing nano-silica particles, the peak of calcium hydroxide and the peak of non-hydrated cement were reduced by nano silica compared to the control mixture**.**

#### TGA

**Figure 6:**
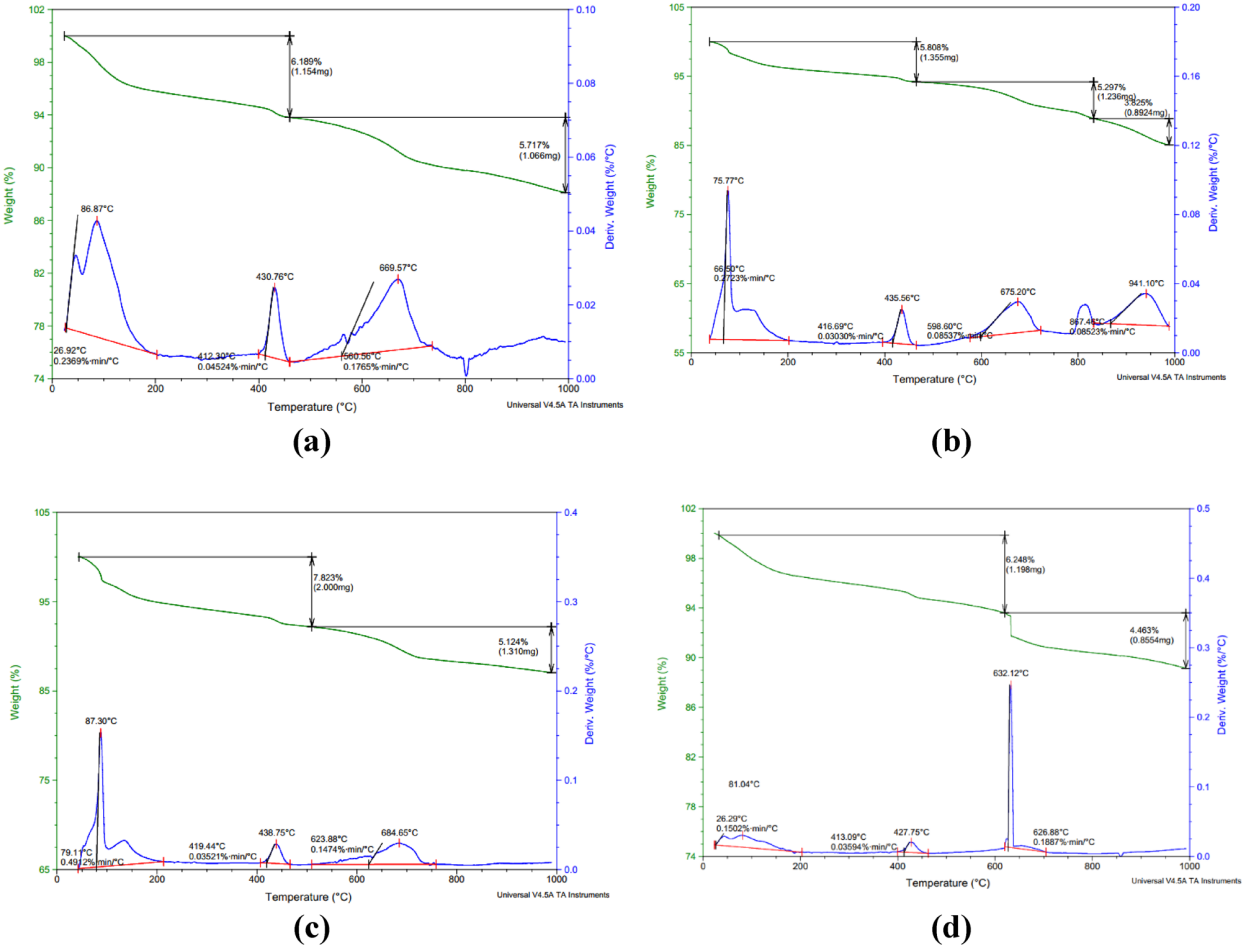
TGA results for (**a**) control specimen; (**b**) specimen with 0% NCL and 1.5% NS; (**c**) specimen with 1% NCL and 0% NS; (**d**) specimen with 1% NCL and 1.5% NS.

The results of the thermogravimetric analysis (TGA) measurements for the control matrix compared to the matrices incorporating nano silica, nano cellulose, and hybrid of both nano materials were represented in Fig. 6. The results showed that the weight loss at the 430 °C due to the water loss that results from the de-hydroxylation of the calcium hydroxide was found to be 6.1%, 5.8%, 7.8%, and 6.2% for the control mix incorporating nano-silica, mix incorporating nano cellulose, and hybrid nano-materials respectively. The results indicate that the least amount of calcium hydroxide was found in the mix incorporating nano-silica. This could be attributed to the high pozzolanic reactivity of nano-silica with the residual calcium hydroxide leading to reducing its content within the matrix. While the highest of them was for the mix incorporating nano cellulose, as a result of the presence of nano cellulose that increases the hydration time increases leading to the formation of more complete and larger Ca(OH)_2_ crystals. Besides, this can also be due to the interaction between cement particles and NCell. The interaction that promote the adsorption of NCell to cement particles, and consequently delay in Cement particles hydration occurs. Several studies have reported similar findings regarding the use of nano-silica and nano-cellulose in cementitious materials^86–88^. For example, a study by Hisseine et al.^86^ reviewed the effect of cellulose nanofibers on the mechanical properties of cementitious composites. These studies support the potential benefits of using nano materials as a partial replacement for cement and improving the properties of cementitious materials^86^.

## Conclusion

The research plan aimed to investigate the effect of the addition of nano-silica particles separately and incorporation with the nano cellulose particles produced from wood sawdust waste at very low dosages on the mechanical and microstructure of Portland cement composite; the results indicated the following:

- The effect of the hybridized nano-silica and nano cellulose improved mechanical properties by increasing the stability and uniformity and improvement of the mix, improving the homogeneity and compactness of the hardened matrices.
- Each nano-material has its unique effect. The high pozzolanic reactivity of nano-silica ensures a minimal amount of calcium hydroxide. While the nano cellulose fibers were found to bridge the calcium silicate hydrate products, arresting the cracks’ propagation.
- The effectiveness of nano cellulose becomes more pronounced at low concentrations. This is related to the high surface area resulting from the less probability of agglomeration that leads to fiber self-assembly and network formation, helping in increasing the mechanical properties and arresting crack propagation.
- The nano cellulose reinforcing effect incorporation with the nano-silica cement composite positively affected the nano cellulose adherence and integration with the cement based matrix as indicated with the microstructural analysis.

## Data Availability

The datasets used and/or analysed during the current study available from the corresponding author on reasonable request.
